# A comparison of five paediatric dosing guidelines for antibiotics

**DOI:** 10.2471/BLT.19.234310

**Published:** 2020-04-28

**Authors:** Shrey Mathur, Charlotte Jackson, Heather Urus, Isabelle Ziarko, Matt Goodbun, Yingfen Hsia, Sally Ellis, Mike Sharland

**Affiliations:** a Paediatric Infectious Diseases Research Group, Institute of Infection and Immunity, St George’s, University of London, Cranmer Terrace, London SW17 0RE, England.; b Global Antibiotic Research and Development Partnership, Drugs for Neglected Diseases initiative, Geneva, Switzerland.

## Abstract

**Objective:**

To compare dosing guidance in the paediatric formularies of high- and middle-income countries for 32 commonly prescribed antibiotics on the World Health Organization’s (WHO’s) 2017 *Model list of essential medicines for children.*

**Methods:**

We identified paediatric antibiotic guidelines that were either widely used internationally or originated from countries in which antibiotic use has increased markedly in recent years (i.e. Brazil, China, India, the Russian Federation and South Africa).

**Findings:**

The study analysis considered five leading antibiotic guidelines: (i) the *Manual of childhood infections: the blue book*; (ii) the *BNF (British national formulary) for children*; (iii) the *Red book^®^: 2018–2021 report of the committee on infectious diseases*; (iv) WHO’s *Pocket book of hospital care for children*; and (v) Indian* National treatment guidelines for antimicrobial use in infectious diseases*. There was marked heterogeneity in the recommended dosing (i.e. daily dose, age dosing bands and dose frequency) for most commonly used antibiotics. The rationale for dosing recommendations was generally unclear.

**Conclusion:**

The pharmacokinetic, pharmacodynamic and clinical evidence supporting paediatric antibiotic dosing, particularly on total doses and on age or weight dosing bands, needs to be improved. Future research should consider whether the variations in guidance identified stem from different clinical disease patterns, varying levels of antibiotic resistance or drug availability rather than historical preferences. Interested global parties could collaborate with WHO’s *Model list of essential medicines* antibiotic working group to develop an evidence-based consensus and identify research priorities.

## Résumé

**Objectif:**

Comparer la posologie des listes de médicaments pédiatriques dans les pays à haut et moyen revenu pour 32 antibiotiques fréquemment prescrits figurant sur l'édition 2017 de la *Liste modèle des médicaments essentiels destinés à l'enfant* de l'Organisation mondiale de la Santé (OMS).

**Méthodes:**

Nous avons identifié plusieurs directives thérapeutiques concernant les antibiotiques à usage pédiatrique, soit largement appliquées à l'échelle internationale, soit émanant de pays où le recours aux antibiotiques a considérablement augmenté ces dernières années (Brésil, Chine, Inde, Fédération de Russie et Afrique du Sud).

**Résultats:**

L'analyse réalisée dans le cadre de l'étude a porté sur cinq recommandations majeures pour le dosage des antibiotiques: (i) le *Manual of childhood infections: the blue book*; (ii) le *BNF (British national formulary) for children*; (iii) le *Red book^®^: 2018–2021 report of the committee on infectious diseases*; (iv) le *Livre de poche pour soins hospitaliers pédiatriques* de l'OMS; et enfin, (v) les *Indian national treatment guidelines for antimicrobial use in infectious diseases*. La posologie recommandée varie beaucoup d'un ouvrage à l'autre (dose journalière, dosage en fonction de la tranche d'âge et fréquence d'administration) pour la plupart des antibiotiques les plus répandus. La logique derrière ces recommandations posologiques n'était généralement pas claire.

**Conclusion:**

Les données pharmacocinétiques, pharmacodynamiques et cliniques justifiant la posologie des antibiotiques destinés aux enfants doivent être améliorées, en particulier pour les doses totales ainsi que le dosage lié à l'âge ou au poids corporel. Les recherches complémentaires devraient déterminer si les variations de recommandations découlent de différents schémas de pathologies cliniques, de niveaux de résistance aux antibiotiques distincts ou de disponibilité des médicaments, ou s'il s'agit plutôt de préférences historiques. Les parties concernées à l'échelle mondiale pourraient collaborer avec le groupe de travail à l'origine de la *Liste modèle des médicaments essentiels destinés à l'enfant* de l'OMS, afin de parvenir à un consensus fondé sur des données concrètes et d'identifier les priorités de recherche.

## Resumen

**Objetivo:**

Comparar las recomendaciones de dosificación en los medicamentos pediátricos de los países de ingresos altos y medios de 32 antibióticos que se recetan con frecuencia en la *Lista modelo de medicamentos pediátricos esenciales *de 2017 de la Organización Mundial de la Salud (OMS).

**Métodos:**

Se identificaron las recomendaciones sobre los antibióticos pediátricos que se utilizaban mucho a nivel internacional o que procedían de países en los que el uso de antibióticos había aumentado notablemente en los últimos años (es decir, Brasil, China, la Federación de Rusia, India y Sudáfrica).

**Resultados:**

El análisis del estudio consideró cinco recomendaciones principales sobre los antibióticos: (i) el *Manual de infecciones infantiles: el libro azul (Manual of childhood infections: the blue book);* (ii) el *Formulario nacional británico (British national formulary, BNF) para niños;* (iii) el *Red book^®^: informe del comité de enfermedades infecciosas de 2018-2021 (Red book^®^: 2018-2021 report of the committee on infectious diseases);* (iv) la *Guía de bolsillo sobre la atención hospitalaria de los niños *de la OMS* (*WHO's* Pocket book of hospital care for children);* y (v) las *Directrices nacionales de tratamiento para el uso de antimicrobianos en las enfermedades infecciosas de la India (Indian national treatment guidelines for antimicrobial use in infectious diseases).* Se observó una marcada heterogeneidad en la dosificación recomendada (es decir, dosis diaria, grupos de dosificación por edad y frecuencia de la dosis) de los antibióticos más utilizados. En general, los motivos de las recomendaciones sobre la dosificación no estaban claros.

**Conclusión:**

Se deben mejorar las evidencias farmacocinéticas, farmacodinámicas y clínicas que respaldan la dosificación de los antibióticos pediátricos, en especial sobre las dosis totales y sobre los grupos de dosificación por edad o peso. Las investigaciones futuras deben considerar si las variaciones en las recomendaciones identificadas se derivan de diferentes patrones clínicos de la enfermedad, de niveles variables de resistencia a los antibióticos o de la disponibilidad de los medicamentos, más que de las preferencias históricas. Las partes interesadas a nivel mundial podrían colaborar con el grupo de trabajo sobre antibióticos de la *Lista modelo de medicamentos esenciales* de la OMS para desarrollar un consenso basado en las evidencias y para identificar las prioridades de investigación.

## ملخص

الغرض مقارنة بين إرشادات الجرعات في تركيبات أدوية الأطفال في البلدان ذات الدخل المرتفع والمتوسط لـ 32 من المضادات الحيوية الموصوفة بشكل شائع في القائمة النموذجية لمنظمة الصحة العالمية (WHO) لعام 2017 للأدوية الأساسية للأطفال.

الطريقة قمنا بتحديد المبادئ التوجيهية للمضادات الحيوية للأطفال، والتي تم إما استخدامها على نطاق واسع بشكل دولي، أو نشأت في البلدان التي زاد فيها استخدام المضادات الحيوية بشكل ملحوظ في السنوات الأخيرة (أي البرازيل والصين والهند والاتحاد الروسي وجنوب إفريقيا).

النتائج ارتكز تحليل الدراسة على خمسة مبادئ توجيهية أساسية للمضادات الحيوية: كتيب أمراض الطفولة: **الكتاب الأزرق؛ و(ب) تركيبات الأدوية الوطنية البريطانية (BNF) للأطفال؛ و(ج) الكتاب الأحمر * (Red Book ^® ^): *تقرير من لجنة الأمراض المعدية للفترة من 2018 إلى 2021؛ و(د) كتاب منظمة الصحة العالمية للجيب عن رعاية الأطفال في المستشفيات؛ و(هـ) المبادئ التوجيهية في العلاج الوطني الهندي لاستخدام مضادات الميكروبات في الأمراض المعدية. كان هناك عدم تجانس ملحوظ في الجرعات الموصى بها (أي الجرعة اليومية، ونطاقات الجرعات العمرية، وتكرار الجرعة) بالنسبة للمضادات الحيوية الأكثر استخداماً. كان السبب المنطقي وراء توصيات الجرعات غير واضح بشكل عام.

الاستنتاج يجب تحسين حركيات الدواء، والديناميكا الدوائية، والأدلة السريرية التي تدعم جرعات المضادات الحيوية للأطفال، وخاصة على الجرعات الكلية وعلى شرائح الجرعات الخاصة بالعمر أو الوزن. يجب أن تضع الأبحاث المستقبلية في الاعتبار ما إذا كانت الاختلافات في المبدأ التوجيهي المحدد تنشأ عن أنماط سريرية مختلفة للأمراض، أو مستويات مختلفة من مقاومة المضادات الحيوية، أو توافر الأدوية، وليست مجرد تفضيلات تاريخية. يمكن للأطراف العالمية المعنية أن تتعاون مع مجموعة عمل المضادات الحيوية في **القائمة النموذجية لمنظمة الصحة العالمية (WHO) للأدوية الأساسية للأطفال، بهدف الوصول إلى إجماع قائم على الأدلة وتحديد أولويات البحث.

## 摘要

**目的:**

旨在比较世界卫生组织 (WHO) 2017 年《儿童基本药物标准清单》中 32 种常用抗生素在中高收入国家和新兴经济国家儿科处方中的用药指南。

**方法:**

我们选定了来自全球范围内广泛使用或近年来抗生素使用量大幅增加的国家（即巴西、俄罗斯、南非、印度和中国）制定的儿科抗生素指南。

**结果:**

这项研究分析考量了五大抗生素指南：(i) *《儿童感染手册：蓝皮书》（Manual of childhood infections: the blue book）；*(ii) *《英国国家儿童处方集》（BNF (British national formulary) for children）*；(iii)《*红皮书：2018–2021 传染病委员会报告》（Red book^®^: 2018–2021 report of the committee on infectious diseases）；*(iv) 世界卫生组织发布的*《儿童医院护理口袋书》（Pocket book of hospital care for children）*以及 (v)*《印度传染病使用抗菌药物国民治疗指南》（Indian national treatment guidelines for antimicrobial use in infectious diseases）。*大多数常用抗生素的推荐剂量（即每日剂量、年龄剂量范围和用药频率）存在明显的异质性。推荐剂量的理论依据一般无法查明。

**结论:**

支持儿科抗生素给药剂量（尤其是总剂量、年龄和体重用药范围）的药代动力学、药效学和临床证据，需要完善。未来的研究应考虑所确定的指南差异是否源于不同的临床疾病模式、不同的抗生素耐药性水平或药物可用性，而非历史偏好。全球有意者可与世界卫生组织基本药物标准清单抗生素工作组合作，形成循证共识并确定研究重点。

## Резюме

**Цель:**

Сравнить руководящие принципы по определению дозировки 32 наиболее часто назначаемых антибиотиков для детей в странах с высоким и средним уровнем дохода с учетом принятого Всемирной организацией здравоохранения (ВОЗ) в 2017 г. *Примерного перечня жизненно важных лекарственных препаратов для детей*.

**Методы:**

Авторы рассмотрели руководящие принципы по применению антибиотиков в педиатрии, которые либо широко использовались в международной практике, либо были разработаны в странах, где в последние годы применение антибиотиков сильно возросло (например, Бразилия, Индия, Китай, Российская Федерация и Южная Африка).

**Результаты:**

В рамках исследования анализировались пять ведущих руководящих принципов по применению антибиотиков: (i) «Руководство по инфекционным заболеваниям у детей: официальный сборник документов» (*Manual of childhood infections: the blue book)*; (ii) «Британский национальный педиатрический формуляр» (*BNF (British national formulary) for children)*; (iii) «Красная книга: отчет комитета по инфекционным заболеваниям за 2018–2021 годы» (*Red book^®^: 2018–2021 report of the committee on infectious diseases)*; (iv) изданный ВОЗ «Карманный справочник по больничному уходу за детьми» (*Pocket book of hospital care for children)*; (v) «Индийские национальные рекомендации по применению антимикробных препаратов при инфекционных заболеваниях» (*Indian national treatment guidelines for antimicrobial use in infectious diseases*). Рекомендуемые дозировки (суточные дозы, диапазоны доз по возрасту и частота приема) для большинства наиболее распространенных антибиотиков сильно различались. Обоснования для рекомендаций по дозировке, как правило, были нечеткими.

**Вывод:**

Необходимо совершенствовать систему фармакокинетических, фармакодинамических и клинических данных, обосновывающих дозировку антибиотиков для детей, в частности в отношении общей дозы, возрастных диапазонов или диапазонов из расчета на массу тела. В будущих исследованиях следует выяснить, являются ли обнаруженные расхождения в рекомендациях скорее следствием различных картин клинических проявлений болезней, неоднородной резистентности к антибиотикам или доступности препаратов, чем желанием следовать исторически сложившимся традициям. Заинтересованные представители мирового сообщества могут сотрудничать с рабочей группой по вопросам применения антибиотиков на основе издаваемого ВОЗ *Примерного перечня жизненно важных лекарственных препаратов для детей* с целью разработки единого мнения, основанного на доказательном подходе, и определения приоритетов исследований.

## Introduction

Global antibiotic consumption increased markedly between 2000 and 2010, with Brazil, China, India, the Russian Federation and South Africa accounting for 76% of the increase.^1^ In response, the World Health Organization (WHO) developed a global action plan on antimicrobial resistance in 2015.^2^ The fourth objective of this plan is to optimize the use of antibiotics. More recently, the classification of antibiotics in the *WHO Model list of essential medicines* has undergone substantial revision.^3^^,^^4^ The new AWaRe classification divides antibiotics into Access, Watch and Reserve antibiotic groups with the aim of encouraging their rational use and optimizing prescribing.^3^ Given this renewed focus on prudent antibiotic use, it is important that national prescribing guidelines are reviewed, any variations between countries are identified and the reasons for those variations are understood. Antimicrobials are the most commonly prescribed class of drugs for children.^5^^–^^9^ Historically, however, paediatric dosing regimens have often been derived from pharmacokinetic data in adults, with the assumption that the relationship between drug exposure and total body weight is linear.^10^^,^^11^ This approach, although clinically widespread, is not supported by solid empirical evidence and may result in neonates and children being exposed to inappropriate systemic drug levels.^12^^,^^13^ The potential impact of inappropriate drug use in children on selection for antimicrobial resistance and the development of toxicity is unknown.

Clinicians’ prescribing practices are often informed by formularies, which recommend antibiotic doses that balance efficacy, toxicity and drivers of antimicrobial resistance. However, recommendations are frequently based on historical practice rather than evidence.^14^ For children, in particular, few data on efficacy, safety and pharmacokinetics are available.^15^^–^^17^ Traditionally, there has been a preference for weight-based dosing strategies in the United States of America, whereas the United Kingdom of Great Britain and Northern Ireland has preferred age-banded dosing and WHO has preferred weight-banded dosing. This lack of standardization has resulted in widely varying recommendations and heterogeneous guidance, which have created ambiguities, especially for inexperienced clinicians.^18^^,^^19^ While there has tended to be some agreement on adult dosing guidance, for example between the National Institute for Health and Care Excellence in the United Kingdom and the Infectious Diseases Society of America in the United States, this has not been the case for children.^20^^,^^21^ Our view is that the international variation in guidance on paediatric dosing is most likely not based on different rates of antimicrobial resistance or clinical disease patterns, but instead reflects historical and cultural practices and the absence of a solid evidence base.

Although previous efforts have been made to compare local paediatric antibiotic guidelines, to the best of our knowledge there has been no detailed comparison of guidance from leading paediatric antibiotic formularies globally.^22^ Consequently, the aim of our study was to compare antibiotic guidance in the paediatric antibiotic formularies of both high-income countries and emerging economies for 32 commonly prescribed antibiotics on the 2017 *WHO Model list of essential medicines for children*.^23^

## Methods

We identified antibiotic guidelines that were either widely used internationally or originated in countries in which antibiotic use has increased markedly in recent years (i.e. Brazil, China, India, the Russian Federation and South Africa).^1^ In particular, we looked for guidance in these countries that had been endorsed by national and international bodies by contacting national coordinators of the Global Antibiotic Resistance, Prescribing and Efficacy among Neonates and Children (GARPEC) network.^24^ Our aim was not to review all existing antibiotic guidance from every country comprehensively or to identify all patient management pathways. Instead, we selected guidelines that included specific paediatric dosing formularies or summaries, namely:

- *Manual of childhood infections: the blue book*, 4th edition, 2016, which is endorsed by the Royal College of Paediatrics and Child Health in the United Kingdom and the European Society of Paediatric Infectious Diseases and is a leading handbook used in Europe;^11^
- *BNF (British national formulary) for children,* 2017, which is a commonly used paediatric reference for prescribing in the United Kingdom;^25^
- *Red book^®^: 2018–2021 report of the committee on infectious diseases, 31st edition*, which is endorsed by the American Academy of Pediatrics Committee on Infectious Diseases;^26^
- *Pocket book of hospital care for children*, second edition, 2013, from WHO, which is part of a series of documents and tools that support the integrated management of childhood illness;^27^ and
- Indian *National treatment guidelines for antimicrobial use in infectious diseases*, 2016, which were developed by the Indian National Centre for Disease Control.^28^

Although we consulted national experts, we were unable to find paediatric antibiotic guidelines from Brazil, China, the Russian Federation or South Africa that were clearly endorsed nationally.

From each publication, we obtained the recommended dosage of all antibiotics listed in section: 6.2 (i.e. antibacterials) of the 2017 *WHO Model list of essential medicines for children*, with the exception of: (i) benzathine benzylpenicillin; (ii) procaine benzylpenicillin; (iii) cefixime; (iv) tigecycline; (v) fosfomycin; (vi) daptomycin; (vii) polymyxins (e.g. colistin); (viii) fourth-generation cephalosporins, with or without a β-lactamase inhibitor (e.g. cefepime); and (ix) fifth-generation cephalosporins, with or without a β-lactamase inhibitor (e.g. ceftaroline). We included levofloxacin in our analysis even though it was not listed in the 2017 model list. We grouped the final selection of 32 antibiotics into the three AWaRe categories and compared dosing recommendations made by the different guidelines.

The *BNF for children*, in general, arranges guidance by route of administration and then by age band. The *Blue book* provides guidance by drug, giving the dose in milligrams per kilogram and the dosing frequency per day. The *Red book*® presents separate tables for neonates and children, with neonatal doses stratified by gestational age and then by postnatal age (where applicable): paediatric recommendations are given as a total daily dose per kilogram with a frequency of administration. The *Pocket book of hospital care for children* presents separate summary tables for neonates and children; guidance on neonatal dosing is given by route of administration and stratified by age into the first week of life and a postnatal age of 2 to 4 weeks. The Indian National Centre for Disease Control guidelines present two dosing summary tables: (i) a dosing guide for commonly used antimicrobial agents; and (ii) drug doses in the paediatric age group. We primarily used information from the first table, as the second table was less complete. However, we consulted the second table and the text to resolve inconsistencies. The Indian guidance did not present a separate table for neonates. 

Where possible, we present doses in mg/kg per day or mg/day along with dosing frequency to enable guidelines to be compared. When dosing guidance was given for specific syndromes or suspected causative organisms, we included those for priority syndromes (i.e. pneumonia, sepsis, acute otitis media, pharyngitis and urinary tract infection) and for severe infection, especially meningitis. We excluded prophylactic doses, loading doses and doses for nonpriority syndromes and for low- or very-low-birthweight infants. In all cases where the guidance provide in summary tables was unclear, we consulted the primary text.

## Results

For all 32 antibiotics studied, there were differences in recommended dosages across the five guidelines (Table 1 ; available at: http://www.who.int/bulletin/volumes/98/6/19-234310). Of all the guidelines, the *Pocket book* gave dosing guidance for the fewest antibiotics reviewed (i.e. 12 of 32); it did not give recommendations for some commonly used Access (e.g. amoxicillin with clavulanic acid, phenoxymethylpenicillin and amikacin) and Watch (e.g. meropenem, piperacillin–tazobactam and vancomycin) antibiotics. In fact, it included guidance on only one antibiotic in the Watch group (i.e. erythromycin) and none in the Reserve group. 

**Table 1:** Dosing recommendations in five widely used, guidelines for 32 commonly prescribed, paediatric antibiotic formulations, 2018

Antibiotic	Guidelines, dosing recommendations, by age group, administration route, dosage, frequency				
	*BNF for children*^a^	*Blue book*^b^	*Red book*® ^c^	*Pocket book*^d^	Indian National Centre for Disease Control^e^
**Amikacin**	**Neonate:** • intravenous, 15 mg/kg per day, q24h; **1 month – 11 years:** • intravenous, 15 mg/kg per day, q12h; **12–17 years:** • intravenous, 15 mg/kg per day, q12h	**Neonate:** • intravenous, 15 mg/kg per day, q36h (PMA: ≤ 28 weeks); • intravenous, 15 mg/kg per day, q24h (PMA: > 28 weeks); **1 month – 18 years:** • intravenous, 15 mg/kg per day, q24h	**≤ 7 days (GA: ≥ 35 weeks):** • intramuscular/intravenous,15 mg/kg per day, q24h; **> 7 days (GA: ≥ 35 weeks):** • intramuscular/intravenous, 18 mg/kg per day, q24h; **≤ 14 days:** • intramuscular/intravenous, 15 mg/kg per day, q48h (GA: < 30 weeks); • intramuscular/intravenous, 15 mg/kg per day, q36h (GA: 30–34 weeks); **> 14–28 days:** • intramuscular/intravenous, 15 mg/kg per day, q24h (GA: < 30 weeks); • intramuscular/intravenous, 15 mg/kg per day, q24h (GA: 30–34 weeks); **> 28 days:** • intramuscular/intravenous, 15–22.5 mg/kg per day, q8h–q12h or q24h	No information	• intravenous, 15–22.5 mg/kg per day, q8h–q12h
**Amoxicillin**	**< 7 days:** • intravenous, 60 mg/kg per day, q12h; **7 days – 1 month:** • oral/intravenous, 90 mg/kg per day, q8h; **> 1–11 months:** • oral, 375 mg/day, q8h; **1–4 years:** • oral, 750 mg/day, q8h; **Child:** • intravenous, 60–90 mg/kg per day, q8h; **5–11 years:** • oral, 1500 mg/day, q8h; **12–17 years:** • oral, 1500 mg/day, q8h	**< 7 days:** • oral/intravenous, 60–120 mg/kg per day, q12h; **7–28 days:** • oral/intravenous, 90–180 mg/kg per day, q8h; **1 month – 18 years:** • oral, 45–90 mg/kg per day, q8h; • intravenous, 90–180 mg/kg per day, q8h	**> 28 days – 11 years:** • oral, 40–45 mg/kg per day, q8h (standard dose); • oral, 80–90 mg/kg per day, q12h (high dose); • oral, 90 mg/kg per day, q12h (acute otitis media); • oral, 50 mg/kg per day, q24h (streptococcal pharyngitis); **≥ 12 years:** • oral, 775 mg/day (extended-release formulation), q24h	• oral, 50 mg/kg per day, q12h; • oral, 80 mg/kg per day, q12h (pneumonia)	• oral, 20–50 mg/kg per day, q6h–q8h
**Amoxicillin with clavulanic acid**	**Neonate:** • oral, 0.75 mL/kg per day (125/31 suspension), q8h; • intravenous, 60 mg/kg per day, q12h; **1–2 months:** • intravenous, 60 mg/kg per day, q12h; **1–11 months:** • oral, 0.75 mL/kg per day (125/31 suspension), q8h; **3 months – 17 years:** • intravenous, 90 mg/kg per day, q8h; **1–5 years:** • oral, 0.75 mL/kg per day (125/31 suspension), q8h; **6–11 years:** • oral, 0.45 mL/kg per day (250/62 suspension), q8h; **12–17 years:** • oral, 750 mL/day (250/62 suspension), q8h	**< 28 days:** • oral, 0.75 mL/kg per day (125/31 suspension), q8h; • intravenous, 60 mg/kg per day, q12h; **1–3 months:** • intravenous, 90 mg/kg per day, q8h; **1 month – 6 years:** • oral, 0.75–1.5 mL/kg per day (125/31 suspension), q8h; **2 months – 18 years:** • oral, 0.3–0.4 mL/kg per day (400/57 suspension), q12h; **3 months – 18 years:** • intravenous, 90–120 mg/kg per day, q6h–q8h; **6–12 years:** • oral, 0.45–0.9 mL/kg per day (250/62 suspension), q8h; **12–18 years:** • oral, 250/125-mg tablet, q8h; • oral, 500/125-mg tablet, q8h (severe infection)	**> 28 days:** • oral, 90 mg/kg per day (14 : 1 formulation), q12h; • oral, 25–45 mg/kg per day (7 : 1 formulation), q12h; • oral, 20–40 mg/kg per day (4 : 1 formulation), q8h	No information	• oral/intravenous, 40 mg/kg per day, q12h
**Ampicillin**	**< 7 days:** • intravenous, 60 mg/kg per day, q12h; **7–20 days:** • oral/intravenous, 90 mg/kg per day, q8h; **21–28 days:** • oral/intravenous, 120 mg/kg per day, q6h; **1 month – 18 years:** • intravenous, 100 mg/kg per day, q6h; **1–11 months:** • oral, 500 mg/day, q6h; **1–4 years:** • oral, 1000 mg/day, q6h; **5–11 years:** • oral, 2000 mg/day, q6h; **12–17 years:** • oral, 2000 mg/day, q6h	**≤ 7 days:** • oral/intravenous, 60–120 mg/kg per day, q12h; **7–21 days:** • oral/intravenous, 90–180 mg/kg per day, q8h; **21 days – 1 month:** • oral/intravenous, 120–240 mg/kg per day, q6h; **1 month – 18 years:** • oral, 60–120 mg/kg per day, q6h; • intravenous, 100 mg/kg per day, q6h; • intravenous, 200 mg/kg per day, q6h (severe infection); • intravenous, 600 mg/kg per day, q4h (meningitis)	**≤ 7 days:** • intramuscular/intravenous, 50 mg/kg per day, q12h (GA: ≤ 34 weeks); • intramuscular/intravenous, 50 mg/kg per day, q8h (GA: > 34 weeks); **> 7–28 days (GA: ≤ 34 weeks):** • intramuscular/intravenous, 75 mg/kg per day, q12h; **> 28 days:** • oral, 50–100 mg/kg per day, q6h; • intramuscular/intravenous, 50–200 mg/kg per day, q6h; • intramuscular/intravenous, 300–400 mg/kg per day, q4h (meningitis or endocarditis)	**≤ 7 days:** • intramuscular/intravenous, 100 mg/kg per day, q12h; **8–28 days:** • intramuscular/intravenous, 150 mg/kg per day, q8h; **> 2 months:** • intramuscular/intravenous, 200 mg/kg per day, q6h	• oral/intravenous, 100–400 mg/kg per day, q6h
**Azithromycin**	**6 months – 17 years:** • oral, 10 mg/kg per day, q24h; • oral, 200 mg/day, q24h (weight: 15–25 kg); • oral, 300 mg/day, q24h (weight: 26–35 kg); • oral, 400 mg/day, q24h (weight: 36–45 kg); • oral, 500 mg/day, q24h (weight: > 45 kg)	**1 month – 12 years:** • oral, 10 mg/kg per day, q24h (weight: < 15 kg); • oral, 200 mg/day, q24h (weight: 15–25 kg); • oral, 300 mg/day, q24h (weight: 26–35 kg); • oral, 400 mg/day, q24h (weight: 36–45 kg); •oral, 500 mg/day, q24h (weight: > 45 kg); **12–18 years:** • oral, 1000 mg/day, q24h	**≤ 28 days:** • intravenous/oral, 10 mg/kg per day, q24h; **> 28 days:** • oral, 5–10 mg/kg per day (immediate-release formulation), q24h; • oral, 60 mg/kg per day (extended-release formulation), q24h	No information	• oral, 10 mg/kg per day, q24h
**Aztreonam**	**< 7 days:** • intravenous, 60 mg/kg per day, q12h; **7 days – 1 month:** • intravenous, 90–120 mg/kg per day, q6h–q8h; **> 1 month – 11 years:** • intravenous, 90–120 mg/kg per day, q6h–q8h; **12–17 years:** • intravenous, 3000 mg/day, q8h	**≤ 7 days:** • intravenous, 60 mg/kg per day, q12h; **7 days – 1 month:** • intravenous, 90–120 mg/kg per day, q6h–q8h; **1 month – 2 years:** • intravenous, 90–120 mg/kg per day, q6h–q8h; **2–18 years:** • intravenous, 90 mg/kg per day, q8h; • intravenous, 200 mg/kg per day, q6h (severe infection)	**≤ 7 days:** • intravenous, 30 mg/kg per day, q12h (GA: < 34 weeks); • intravenous, 30 mg/kg per day, q8h (GA: ≥ 34 weeks); **> 7–28 days:** • intravenous, 30 mg/kg per day, q8h (GA: < 34 weeks); • intravenous, 30 mg/kg per day, q6h (GA: ≥ 34 weeks); **> 28 days:** • intramuscular/intravenous, 90–120 mg/kg per day, q6h–q8h	No information	• intravenous, 30–120 mg/kg per day, q6h–q8h
**Benzylpenicillin (penicillin G)**	**< 7 days:** • intramuscular/intravenous, 50 mg/kg per day, q12h; **7–28 days:** • intramuscular/intravenous, 75 mg/kg per day, q8h; **Child:** • intramuscular/intravenous, 100 mg/kg per day, q6h	**≤ 7 days:** • intravenous, 50 mg/kg per day, q12h; **7–28 days:** • intravenous, 75 mg/kg per day, q8h; **1 month:** • intravenous, 150 mg/kg per day, q8h (severe infection); • intravenous, 225 mg/kg per day, q8h (meningitis); **1 month – 18 years:** • intravenous, 100 mg/kg per day, q6h; • intravenous, 200–300 mg/kg per day, q4h–q6h (severe infection)	**≤ 7 days:** • intravenous/intramuscular, 50 000 IU/kg per day (penicillin G aqueous), q12h; • intramuscular, 50 000 IU/kg per day (penicillin G procaine), q24h; **> 7–28 days:** • intravenous, 150 000 IU/kg per day, q8h; **> 28 days:** • intramuscular/intravenous, 100 000–300 000 IU/kg per day, q4h–q6h; • intramuscular/intravenous, 300 000–400 000 IU/kg per day, q4h (meningitis)	**≤ 7 days:** • 100 000 IU/kg per day, q12h; **8 days – 2 months:** • 200 000 IU/kg per day, q6h; **> 2 months:** • intramuscular/intravenous, 200 000 IU/kg per day, q6h; • intramuscular/intravenous, 400 000 IU/kg per day, q6h (meningitis)	• oral, 200 000 IU/kg per day, q6h; • intravenous, 200 000–400 000 IU/kg per day, q6h
**Cefalexin**	**< 7 days:** • oral, 50 mg/kg per day, q12h; **7–20 days:** • oral, 75 mg/kg per day, q8h; **21–28 days:** • oral, 100 mg/kg per day, q6h; **1–11 months:** • oral, 25 mg/kg per day, q12h; **1–4 years:** • oral, 25 mg/kg per day, q12h; **5–11 years:** • oral, 25 mg/kg per day, q12h; **12–17 years:** • oral, 1000–1500 mg/day, q8h–q12h	**≤ 7 days:** • oral, 50 mg/kg per day, q12h; **7–21 days:** • oral, 75 mg/kg per day, q8h; **21–28 days:** • oral, 100 mg/kg per day, q6h; **1 month – 18 years:** • oral, 25–50 mg/kg per day, q6h	**> 28 days:** • oral, 25–50 mg/kg per day, q12h; • oral, 75–100 mg/kg per day, q6h–q8h (bone or joint infection)	• oral, 50 mg/kg per day, q6h	• oral, 30–40 mg/kg per day, q8h
**Cefazolin**	No information	No information	**≤ 7 days (GA: ≥ 32 weeks):** • intravenous/intramuscular, 50 mg/kg per day, q12h; **> 7 days (GA: ≥ 32 weeks):** • intravenous/intramuscular, 50 mg/kg per day, q8h; **< 14 days (GA: < 32 weeks):** • intravenous/intramuscular, 50 mg/kg per day, q12h; **≥ 14 days (GA: < 32 weeks):** • intravenous/intramuscular, 50 mg/kg per day, q8h; **> 28 days:** • intramuscular/intravenous, 25–75 mg/kg per day, q8h; • intramuscular/intravenous, up to 150 mg/kg per day, q6h–q8h (bone or joint infection)	No information	• intravenous, 100 mg/kg per day, q6h–q8h
**Cefotaxime**	**< 7 days:** • intramuscular/intravenous, 50 mg/kg per day, q12h; • intramuscular/intravenous, 100 mg/kg per day, q12h (meningitis); **7–20 days:** • intramuscular/intravenous, 75 mg/kg per day, q8h; • intramuscular/intravenous, 150 mg/kg per day, q8h (meningitis); **21–28 days:** • intramuscular/intravenous, 75–100 mg/kg per day, q6h–q8h; • intramuscular/intravenous, 150–200 mg/kg per day, q6h–q8h (meningitis); **Child:** • oral, 100–150 mg/kg per day, q8h–q12h; • intramuscular/intravenous, 200–300 mg/kg per day, q8h–q12h (meningitis)	**≤ 7 days:** • intravenous, 50–100 mg/kg per day, q12h; **7–21 days:** • intravenous, 75–150 mg/kg per day, q8h; **21–28 days:** • intravenous, 100–200 mg/kg per day, q6h; **1 month – 18 years:** • intravenous, 150–200 mg/kg per day, q6h–q8h	**≤ 7 days (GA: ≥ 32 weeks):** • intravenous/intramuscular, 50 mg/kg per day, q12h; **> 7 days (GA: ≥ 32 weeks):** • intravenous/intramuscular, 50 mg/kg per day, q8h; **< 14 days (GA: < 32 weeks):** • intravenous/intramuscular, 50 mg/kg per day, q12h; **≥ 14 days (GA: < 32 weeks):** • intravenous/intramuscular, 50 mg/kg per day, q8h; **> 28 days:** • intramuscular/intravenous, 150–180 mg/kg per day, q8h; • intramuscular/intravenous, 200–225 mg/kg per day, q6h (meningitis)	**< 7 days:** • intravenous, 150 mg/kg per day, q8h; **2–4 weeks:** • intravenous, 200 mg/kg per day, q6h; **> 2 months:** • intravenous, 200 mg/kg per day, q6h	• intravenous, 100 mg/kg per day, q6h–q8h; • intravenous, 200 mg/kg per day, q6h (meningitis)
**Ceftazidime**	**< 7 days:** • intravenous, 25 mg/kg per day, q24h; **7–20 days:** • intravenous, 50 mg/kg per day, q12h; **21–28 days:** • intravenous, 75 mg/kg per day, q8h; **Child:** • intravenous, 75 mg/kg per day, q8h	**≤ 7 days:** • intravenous, 25–50 mg/kg per day, q24h; **7–21 days:** • intravenous, 50–100 mg/kg per day, q12h; **21–28 days:** • intravenous, 75–150 mg/kg per day, q8h; **1 month – 18 years:** • intravenous, 25–50 mg/kg per day, q24h	**≤ 7 days (GA: ≥ 32 weeks):** • intravenous/intramuscular, 50 mg/kg per day, q12h; **> 7 days (GA: ≥ 32 weeks):** • intravenous/intramuscular, 50 mg/kg per day, q8h; **< 14 days (GA: < 32 weeks):** • intravenous/intramuscular, 50 mg/kg per day, q12h; **≥ 14 days (GA: < 32 weeks):** • intravenous/intramuscular, 50 mg/kg per day, q8h; **> 28 days:** • intramuscular/intravenous, 90–150 mg/kg per day, q8h; • intramuscular/intravenous, 200–300 mg/kg per day, q8h (severe *Pseudomonas* infection)	No information	• intramuscular/intravenous, 75–100 mg/kg per day, q8h; • intravenous, 100 mg/kg per day, q8h (meningitis)
**Ceftriaxone**	**< 15 days:** • intravenous, 20–50 mg/kg per day, q24h; **15–28 days:** • intravenous, 50–80 mg/kg per day, q24h; **1 month – 11 years (weight: < 50 kg):** • intramuscular/intravenous, 50–80 mg/kg per day, q24h; **9–11 years (weight: ≥ 50 kg):** • intramuscular/intravenous, 1000–2000 mg/day, q24h; **12–17 years:** • intramuscular/intravenous, 1000–2000 mg/day, q24h	**< 28 days:** • intramuscular/intravenous, 25–50 mg/kg per day, q24h; **1 month – 18 years:** • intramuscular/intravenous, 50–80 mg/kg per day, q24h; • intramuscular/intravenous, 80 mg/kg per day, q24h (meningitis)	**> 28 days:** • intramuscular/intravenous, 50–75 mg/kg per day, q24h; • intramuscular/intravenous, 100 mg/kg per day, q12h–q24h (severe infection); • intramuscular, 50 mg/kg per day, q24h (acute otitis media)	**< 2 months:** • intramuscular/intravenous, 100 mg/kg per day, q12h–q24h; **> 2 months:** • intravenous, 80 mg/kg per day, q24h; • intramuscular/intravenous, 100 mg/kg per day, q12h (meningitis)	• intravenous, 50–100 mg/kg per day, q12h; • intravenous, 100 mg/kg per day, q12h (meningitis)
**Cefuroxime**	**< 7 days:** • intravenous, 50 mg/kg per day, q12h; **7–20 days:** • intravenous, 75 mg/kg per day, q8h; **21–28 days:** • intravenous, 100 mg/kg per day, q6h; **Child:** • intramuscular/intravenous, 60 mg/kg per day, q8h; **3 months – 1 year:** • oral, 20 mg/kg per day, q12h; **2–11 years:** • oral, 30 mg/kg per day, q12h; **12–17 years:** • oral, 500 mg/day, q12h	**≤ 7 days:** • intravenous, 50–100 mg/kg per day, q12h; **7–21 days:** • intravenous, 75–150 mg/kg per day, q8h; **21–28 days:** • intravenous, 100–200 mg/kg per day, q6h; **1 month – 18 years:** • intravenous, 60–240 mg/kg per day, q6h–q8h; **3 months – 18 years:** • oral, 20–30 mg/kg per day, q12h	**≤ 7 days (GA: ≥ 32 weeks):** • intravenous/intramuscular, 50 mg/kg per day, q12h; **> 7 days (GA: ≥ 32 weeks):** • intravenous/intramuscular, 50 mg/kg per day, q8h; **< 14 days (GA: < 32 weeks):** • intravenous/intramuscular, 50 mg/kg per day, q12h; **≥ 14 days (GA: < 32 weeks):** • intravenous/intramuscular, 50 mg/kg per day, q8h; **> 28 days:** • oral, 20–30 mg/kg per day (cefuroxime axetil), q12h; • oral, ≤ 100 mg/kg per day (cefuroxime axetil), q8h (bone or joint infection); • intravenous/intramuscular, 100–150 mg/kg per day, q8h	No information	• oral, 20–30 mg/kg per day, q12h; • intravenous, 75–100 mg/kg per day, q8h
**Chloramphenicol**	**< 14 days:** • intravenous, 25 mg/kg per day, q12h; **14–28 days:** • intravenous, 25–50 mg/kg per day, q6h–q12h; **Child:** • oral/intravenous, 50 mg/kg per day, q6h; • oral/intravenous, 50 mg/kg per day, q6h (meningitis)	**≤ 14 days:** • intravenous, 25 mg/kg per day, q12h; **14–28 days:** • intravenous, 37.5–50 mg/kg per day, q6h–q8h; **1 month – 18 years:** • intravenous, 50–100 mg/kg per day, q6h	**> 28 days:** • intravenous, 50–100 mg/kg per day, q6h	• oral, 75 mg/kg per day, q8h; • intravenous, 100 mg/kg per day, q6h (meningitis)	**> 3 months:** • oral/intravenous, 75–100 mg/kg per day, q6h
**Ciprofloxacin**	**Neonate:** • oral, 30 mg/kg per day, q12h; • intravenous, 20 mg/kg per day, q12h; **Child:** • oral, 40 mg/kg per day, q12h; • intravenous, 30 mg/kg per day, q8h	**< 28 days:** • oral, 20–30 mg/kg per day, q12h; • intravenous, 12–20 mg/kg per day, q12h; **1 month – 18 years:** • oral, 30 mg/kg per day, q12h; • intravenous, 20–30 mg/kg per day, q8h–q12h	**> 28 days:** • oral, 20–40 mg/kg per day, q12h; • intravenous, 20–30 mg/kg per day, q8h–q12h	• oral, 20–40 mg/kg per day, q12h	• oral/intravenous, 20–30 mg/kg per day, q12h
**Clarithromycin**	**Neonate:** • oral, 15 mg/kg per day, q12h; **1 month – 11 years:** • oral, 15 mg/kg per day, q12h (weight: < 8 kg); • oral, 125 mg/day, q12h (weight: 8–11 kg); • oral, 250 mg/day, q12h (weight: 12–19 kg); • oral, 375 mg/day, q12h (weight: 20–29 kg); • oral, 500 mg/day, q12h (weight: 30–40 kg); • intravenous, 15 mg/kg per day, q12h; **12–17 years:** • oral, 500 mg/day, q12h; • intravenous, 1000 mg/day, q12h	**< 28 days:** • oral, 15 mg/kg per day, q12h; • intravenous, 15 mg/kg per day, q12h; **1 month – 18 years:** • oral, 15–30 mg/kg per day, q12h; • intravenous, 15 mg/kg per day, q12h	**> 28 days:** • oral, 15 mg/kg per day, q12h	No information	• oral/intravenous, 15 mg/kg per day, q12h
**Clindamycin**	**< 14 days:** • oral, 9–18 mg/kg per day, q8h; **14 days – 1 month:** • oral, 12–24 mg/kg per day, q6h; **1 month – 17 years:** • oral, 12–24 mg/kg per day, q6h; • intramuscular/intravenous, 15–25 mg/kg per day, q6h	**< 14 days:** • oral, 9–18 mg/kg per day, q8h; **14 days – 1 month:** • oral, 12–24 mg/kg per day, q6h; **1 month – 18 years:** • oral, 24 mg/kg per day, q6h; • intravenous, 24–40 mg/kg per day, q6h	**PMA: ≤ 32 weeks:** • intravenous/oral, 5 mg/kg per day, q8h; **PMA: 33–40 weeks:** • intravenous/oral, 7 mg/kg per day, q8h; **PMA: > 40 weeks:** • intravenous/oral, 9 mg/kg per day, q8h; **> 28 days:** • oral, 10–25 mg/kg per day, q8h; • oral, 30–40 mg/kg per day, q6h–q8h (acute otitis media or community-associated, methicillin-resistant *Staphylococcus aureus*); • intramuscular/intravenous, 20–40 mg/kg per day, q6h–q8h	No information	• oral/intravenous, 40–60 mg/kg per day, q6h–q8h
**Cloxacillin, dicloxacillin or flucloxacillin**	**< 7 days:** • oral/intravenous, 50 mg/kg per day, q12h; **7–20 days:** • oral/intravenous, 75 mg/kg per day, q8h; **21–28 days:** • oral/intravenous, 100 mg/kg per day, q6h; **1 month – 1 year:** • oral, 250–500 mg/day, q6h; **2–9 years:** • oral, 500–1000 mg/day, q6h; **10–17 years:** • oral, 1000–2000 mg/day, q6h; **Child:** • intramuscular/intravenous, 50–100 mg/kg per day, q6h	**< 7 days:** • oral/intravenous, 50 mg/kg per day, q12h; • intravenous, 100 mg/kg per day, q12h (severe infection); **7–21 days:** • oral/intravenous, 75 mg/kg per day, q8h; • intravenous, 150 mg/kg per day, q8h (severe infection); **> 21–28 days:** • oral, 100 mg/kg per day, q6h; • intravenous, 200 mg/kg per day, q6h (severe infection); **1 month – 18 years:** • oral, 100 mg/kg per day, q6h; • intravenous, 100–200 mg/kg per day, q6h	No information	**< 7 days:** • intravenous, 50–100 mg/kg per day, q12h; **2–4 weeks:** • intravenous, 75–150 mg/kg per day, q8h; **> 2 months:** • intramuscular/intravenous, 100–200 mg/kg per day, q6h	• oral, 50–100 mg/kg per day, q6h–q8h; • intravenous, 100–200 mg/kg per day, q6h
**Doxycycline**	**12–17 years:** • oral, 100 mg/day, q24h	**12–18 years:** • oral, 200 mg/day, q12h	**> 28 days:** • oral/intravenous, 2.2–4.4 mg/kg per day, q12h	No information	No information
**Erythromycin**	**< 1 month:** • oral, 50 mg/kg per day, q6h; • intravenous, 40–50 mg/kg per day, q6h; **1 month – 1 year:** • oral, 500 mg/day, q6h–q12h; **2–7 years:** • oral, 1000 mg/day, q6h–q12h; **8–17 years:** • oral, 1000–2000 mg/day, q6h–q12h; **Child:** • intravenous, 50 mg/kg per day, q6h	**< 28 days:** • oral, 50 mg/kg per day, q6h; • intravenous, 40–50 mg/kg per day, q6h; **1 month – 18 years:** • oral, 50–100 mg/kg per day, q6h; • intravenous, 50 mg/kg per day, q6h	**≤ 28 days:** • intravenous/oral, 10 mg/kg per day, q6h; **> 28 days:** • oral, 40–50 mg/kg per day, q6h–q8h; • intravenous, 20 mg/kg per day, q6h	• oral, 50 mg/kg per day, q6h	• oral, 40 mg/kg per day, q6h
**Gentamicin**	**1 month – 11 years:** • intramuscular/intravenous, 7.5 mg/kg per day, q8h; **Child:** • intravenous, 7 mg/kg per day, q24h; **12–17 years:** • intramuscular/intravenous, 6 mg/kg per day, q8h	**Neonate:** • intravenous, 5 mg/kg per dose, q36h (CGA: < 32 weeks); • intravenous, 5 mg/kg per day, q24h (CGA: > 32 weeks); **1 month – 18 years:** • intravenous, 7 mg/kg per day, q24h	**≤ 7 days (GA: ≥ 35 weeks):** • intramuscular/intravenous, 4 mg/kg per day, q24h; **> 7 days (GA: ≥ 35 weeks):** • intramuscular/intravenous, 5 mg/kg per day, q24h; **≤ 14 days:** • intramuscular/intravenous, 5 mg/kg per day, q48h (GA: < 30 weeks); • intramuscular/intravenous, 5 mg/kg per day, q36h (GA: 30–34 weeks); **> 14 days:** • intramuscular/intravenous, 5 mg/kg per day, q36h (GA: < 30 weeks); • intramuscular/intravenous, 5 mg/kg per day, q24h (GA: 30–34 weeks); **> 28 days:** • intramuscular/intravenous, 6–7.5 mg/kg per day, q8h; • intramuscular/intravenous, 5–7.5 mg/kg per day, q24h	**7 days:** • intramuscular/intravenous, 5 mg/kg per day, q24h; **2–4 weeks:** • intramuscular/intravenous, 7.5 mg/kg per day, q24h; **> 2 months:** • intramuscular/intravenous, 7.5 mg/kg per day, q24h	• intramuscular/intravenous, 5–7.5 mg/kg per day, q8h–q12h
**Imipenem with cilastatin**	**< 7 days:** • intravenous, 40 mg/kg per day, q12h; **7–20 days:** • intravenous, 60 mg/kg per day, q8h; **21–28 days:** • intravenous, 80 mg/kg per day, q6h; **1–2 months:** • intravenous, 80 mg/kg per day, q6h; **3 months – 17 years:** • intravenous, 60 mg/kg per day, q6h	**≤ 7 days:** • intravenous, 30 mg/kg per day, q12h; **7–21 days:** • intravenous, 45 mg/kg per day, q8h; **21–28 days:** • intravenous, 60 mg/kg per day, q6h; **1–3 months:** • intravenous, 60 mg/kg per day, q8h; **3 months – 18 years:** • intravenous, 60 mg/kg per day, q6h	**≤ 28 days:** • intravenous, 25 mg/kg per day, q12h (PNA: ≤ 7 days); • intravenous, 25 mg/kg per day, q8h (PNA: > 7 days); **> 28 days:** • intravenous, 60–100 mg/kg per day, q6h	No information	**< 3 months:** • oral/intravenous, 100 mg/kg per day, q6h; **> 3 months:** • oral/intravenous, 60–100 mg/kg per day, q6h
**Levofloxacin**	No information	No information	**≥ 6 months:** • intravenous/oral, 16 mg/kg per day, q12h (weight: < 50 kg); • intravenous/oral, 500 mg/day, q24h (weight: > 50 kg)	No information	**6 months – 5 years:** • NRS, 20 mg/kg per day, q12h; **> 5 years:** • NRS, 10 mg/kg per day, q24h
**Linezolid**	**< 7 days:** • oral/intravenous, 20 mg/kg per day, q12h; **7–28 days:** • oral/intravenous, 30 mg/kg per day, q8h; **1 month – 11 years:** • oral/intravenous, 30 mg/kg per day, q8h; **12–17 years:** • oral/intravenous, 1200 mg/day, q12h	**≤ 7 days:** • oral/intravenous, 20 mg/kg per day, q12h; **> 7–28 days:** • oral/intravenous, 30 mg/kg per day, q8h; **1 month – 12 years:** • oral/intravenous, 30 mg/kg per day, q8h; **> 12–18 years:** • oral/intravenous, 1200 mg/day, q12h	**≤ 7 days:** • intravenous/oral, 10 mg/kg per day, q12h (GA: < 34 weeks); • intravenous/oral, 10 mg/kg per day, q8h (GA: ≥ 34 weeks); **> 7 days:** • intravenous/oral, 10 mg/kg per day, q8h (GA: < 34 weeks); • intravenous/oral, 10 mg/kg per day, q8h (GA: ≥ 34 weeks); **28 days – 11 years:** • oral/intravenous, 30 mg/kg per day, q8h; **> 11 years:** • oral/intravenous, 1200 mg/day, q12h	No information	• oral/intravenous, 30–40 mg/kg per day, q6h–q8h
**Meropenem**	**< 7 days:** • intravenous, 40 mg/kg per day, q12h; **7–28 days:** • intravenous, 60 mg/kg per day, q8h; **1 month – 11 years:** • intravenous, 30–60 mg/kg per day, q8h (weight: < 50 kg); • intravenous, 1500–3000 mg/day, q8h (weight: ≥ 50kg); **12–17 years:** • intravenous, 1500–3000 mg/day, q8h	**≤ 7 days:** • intravenous, 80 mg/kg per day, q12h; **> 7–28 days:** • intravenous, 120 mg/kg per day, q8h; **1 month – 12 years:** • intravenous, 30–120 mg/kg per day, q8h	**< 14 days:** • intravenous, 20 mg/kg per day, q12h (GA: < 32 weeks); • intravenous, 20 mg/kg per day, q8h (GA: ≥ 32 weeks); **≥ 14 days:** • intravenous, 20 mg/kg per day, q8h (GA: < 32 weeks); • intravenous, 30 mg/kg per day, q8h (GA: ≥ 32 weeks); **> 28 days:** • intravenous, 60 mg/kg per day, q8h; • intravenous, 120 mg/kg per day, q8h (meningitis)	No information	**> 3 months:** • intravenous, 60 mg/kg per day, q8h
**Metronidazole**	**Neonate:** • intravenous, 7.5 mg/kg per day, q24h (CGA: < 26 weeks); • intravenous, 15 mg/kg per day, q12h (CGA: 26–34 weeks); • intravenous, 22.5 mg/kg per day, q8h (CGA: ≥ 34 weeks); **1 month:** • oral, 15 mg/kg per day, q12h; • intravenous, 22.5 mg/kg per day, q8h; **> 1–11 month:** • rectal, 375 mg/day, q8h; **2 months – 11 years:** • oral, 22.5 mg/kg per day, q8h; **2 months – 17 years:** • intravenous, 22.5 mg/kg per day, q8h; **1–4 years:** • rectal, 750 mg/day, q8h; **5–9 years:** • rectal, 1500 mg/day, q8h; **10–17 years:** • rectal, 3000 mg/day, q8h; **12–17 years:** • oral, 1200 mg/day, q8h	**< 28 days:** • oral/intravenous, 15 mg/kg per day, q12h; **1 month – 18 years:** • oral/intravenous, 22.5 mg/kg per day, q8h	**≤ 28 days:** • intravenous, 7.5 mg/kg per day, q12h (PMA: ≤ 34 weeks); • intravenous, 7.5 mg/kg per day, q8h (PMA: 35–40 weeks); • intravenous, 10 mg/kg per day, q8h (PMA: > 40 weeks); **> 28 days:** • oral, 15–50 mg/kg per day, q8h; • intravenous, 22.5–40 mg/kg per day, q6h–q8h	• oral, 22.5 mg/kg per day, q8h	• oral/intravenous, 22.5 mg/kg per day, q8h
**Nitrofurantoin**	**3 months – 11 years:** • oral, 3 mg/kg per day, q6h; **12–17 years:** • oral, 200 mg/day, q6h (q12h with modified-release formulation)	**3 months – 18 years:** • oral, 4 mg/kg per day, q6h	**> 28 days:** • oral, 5–7 mg/kg per day, q6h	No information	• oral, 8 mg/kg per day, q12h
**Phenoxymethylpenicillin (penicillin V)**	**1–11 months:** • oral, 250 mg/day, q6h; **1–5 years:** • oral, 500 mg/day, q6h; **6–11 years:** • oral, 1000 mg/day, q6h; **12–17 years:** • oral, 2000 mg/day, q6h	**1 month – 18 years:** • oral, 60 mg/kg per day, q6h	**> 28 days:** • oral, 25–50 mg/kg per day, q6h	No information	• oral, 20–50 mg/kg per day, q6h
**Piperacillin–tazobactam**	**< 1 month:** • intravenous, 270 mg/kg per day, q8h; **1 month – 11 years:** • intravenous, 270–360 mg/kg per day, q6h–q8h; **12–17 years:** • intravenous, 13 500 mg/day, q8h	**< 28 days:** • intravenous, 270 mg/kg per day, q8h; **1 month – 18 years:** • intravenous, 270–360 mg/kg per day, q6h–q8h	**≤ 28 days:** • intravenous, 100 mg/kg per day, q8h (PMA: ≤ 30 weeks); • intravenous, 80 mg/kg per day, q6h (PMA: > 30 weeks); **> 28 days:** • intravenous, 240–300 mg/kg per day, q6h–q8h	No information	• intravenous, 200–400 mg/kg per day, q6h–q8h
**Trimethoprim**	**< 1 month:** • oral, 2–4 mg/kg per day, q12h; **4 weeks – 11 years** • oral, 8 mg/kg per day, q12h; **12–17 years:** • oral, 400 mg/day, q12h	**< 28 days:** • oral, 2–4 mg/kg per day, q12h; **1 month – 18 years:** • oral, 8 mg/kg per day, q12h	No information	No information	No information
**Trimethoprim–sulfamethoxazole**	**6 weeks – 5 months:** • oral, 240 mg/day, q12h; **6 weeks – 17 years:** • intravenous, 36 mg/kg per day, q12h; • intravenous, 54 mg/kg per day, q12h (severe infection); **6 months – 5 years:** • oral, 480 mg/day, q12h; **6 years – 11 years:** • oral, 960 mg/day, q12h; **12–17 years:** • oral, 1920 mg/day, q12h	**6 weeks – 18 years:** • oral, 48 mg/kg per day, q12h	**> 28 days:** • oral/intravenous, 8–10 mg/kg per day, q12h	**≤ 1 month:** • oral, 4 mg/kg per day of trimethoprim and 20 mg/kg per day of sulfamethoxazole, q12h; **> 2 months:** • oral, 8 mg/kg per day of trimethoprim and 40 mg/kg per day of sulfamethoxazole, q12h	• oral, 5–10 mg/kg per day, q12h
**Vancomycin**	**Neonate:** • intravenous, 15 mg/kg per day, q24h (CGA: < 29 weeks); • intravenous, 30 mg/kg per day, q12h (CGA: 29–35 weeks); • intravenous, 45 mg/kg per day, q8h (CGA: > 35 weeks); **Child:** • intravenous, 45 mg/kg per day, q8h	**Neonate:** • intravenous, 15 mg/kg per day, q24h (PMA: < 29 weeks); • intravenous, 30 mg/kg per day, q12h (PMA: 29–35 weeks); • intravenous, 45 mg/kg per day, q8h (PMA: > 35 weeks); **1 month – 18 years:** • intravenous, 45 mg/kg per day, q8h	**< 28 days (GA: ≤ 28 weeks):** • NRS, 15 mg/kg per day, q12h (Cr_S_: < 0.5 mg/dL); • NRS, 20 mg/kg per day, q24h (Cr_S_: 0.5–0.7 mg/dL); • NRS, 15 mg/kg per day, q24h (Cr_S_: 0.8–1 mg/dL); • NRS, 10 mg/kg per day, q24h (Cr_S_: 1.1–1.4 mg/dL); • NRS, 15 mg/kg per day, q48h (Cr_S_: > 1.4 mg/dL); **< 28 days (GA: > 28 weeks):** • NRS, 15 mg/kg per day, q12h (Cr_S_: < 0.7mg/dL); • NRS, 20 mg/kg per day, q24h (Cr_S_: 0.7–0.9 mg/dL); • NRS, 15 mg/kg per day, q24h (Cr_S_: 1–1.2 mg/dL); • NRS, 10 mg/kg per day, q24h (Cr_S_: 1.3–1.6 mg/dL); • NRS, 15 mg/kg per day, q48h (Cr_S_: > 1.6 mg/dL); **> 28 days:** • intravenous, 45–60 mg/kg per day, q6h–q8h; • oral, 40 mg/kg per day, q6h	No information	• intravenous, 40–60 mg/kg per day, q6h–q8h

CGA: corrected gestational age; Cr_S_: serum creatinine; GA: gestational age; IU: international unit; NRS: no route of administration specified; PMA: postmenstrual age; PNA: postnatal age; q6h: every 6 hours; q8h: every 8 hours; q12h: every 12 hours; q24h: every 24 hours; q36h: every 36 hours; q48h: every 48 hours.

^a^ *BNF (British national formulary) for children*.^25^

^b^ *Manual of childhood infections: the blue book*, 4th edition.^11^

^c^ *Red book®: 2018–2021 report of the committee on infectious diseases*, 31st edition.^26^

^d^ *Pocket book of hospital care for children*, second edition.^27^

^e^ *National treatment guidelines for antimicrobial use in infectious diseases*.^28^

The number of dosing bands varied across guidelines, with the *BNF for children* tending to have the most and the *Pocket book* and the Indian National Centre for Disease Control guidelines having the fewest. The rationale for choosing different weight or age bands was not apparent, pharmacokinetic evidence was neither cited nor, if available, explained.^29^

**Fig. 1:**
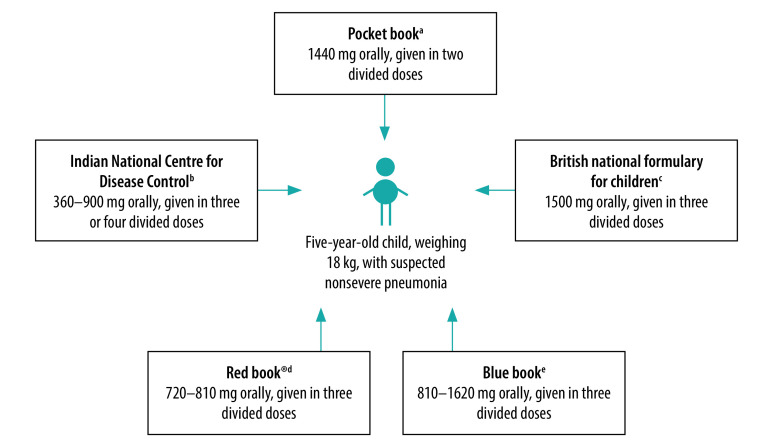
Oral amoxicillin doses recommended by five guidelines for an 18-kg, 5-year-old child with suspected nonsevere pneumonia, 2018

^a^ *Pocket book of hospital care for children*, second edition.^27^

^b^ *National treatment guidelines for antimicrobial use in infectious diseases*.^28^

^c^ *BNF (British national formulary) for children*.^25^

^d^ *Red book®: 2018–2021 report of the committee on infectious diseases*, 31st edition.^26^

^e^ *Manual of childhood infections: the blue book*, 4th edition.^11^

There was a considerable variation in recommended doses for each antibiotic. For example, Fig. 1 illustrates that the recommended dose of oral amoxicillin for a 5-year-old child weighing 18 kg with suspected nonsevere pneumonia varied from 360 mg per day (using the lower end of the Indian National Centre for Disease Control guidance of 20 mg/kg per day) to 1620 mg per day (using the upper end of the *Blue book* guidance of 90 mg/kg per day). Moreover, the five guidelines all proposed different age ranges for dosing and the suggested dosing interval was 6, 8 or 12 hours.

## Discussion

We found marked differences between the guidelines reviewed on paediatric dosing recommendations for 32 commonly prescribed antibiotics. There were differences in the age basis for dosing (e.g. the use of postnatal age alone or in combination with weight or gestational age), the frequency of administration and the total daily dose. Although some of this heterogeneity may reflect differences between settings in epidemiology, risk factors, causative organisms (e.g. due to different vaccination policies) or patterns of antibiotic resistance and may therefore be appropriate, other variations (e.g. the use of different age or weight dosing bands) can create confusion. In many instances, it is difficult to discern the rationale behind the dosing recommendations, whether they are derived from the accompanying summary of product characteristics, academic publications or expert consensus. Several national paediatric formularies, such as the Dutch, Italian and Spanish formularies, have been developed, but have been criticized for not presenting the underlying evidence or for referring to outdated paediatric dosing handbooks.^30^^,^^31^ However, the Dutch formulary has clearly tried to address this issue.^32^

Variations between guidelines may partly be due to a lack of robust evidence on the best treatment. In addition, defining optimal drug dosing is more complex for children than adults: antibiotic doses must be adapted to maturational changes in pharmacokinetics and consider changes in pharmacodynamics, yet still be simple and pragmatic. However, few studies on antibiotic pharmacokinetics or effectiveness have been performed in children.^17^^,^^33^ Further, most of the guidelines we considered did not explicitly reference the evidence underlying their recommendations. The *Blue book* alone indicated the strength of the recommendations for each individual dose but did not present the supporting evidence. Moreover, the pharmacokinetic data underpinning many older off-patent antibiotics are very limited and were based on studies that were not conducted in accordance with recent standards.^34^

A few strategic trials in children have examined high- and low-dose strategies and have reported efficacy and toxicity outcomes. However, these studies have used widely varying inclusion and exclusion criteria and end-points, which makes data synthesis between trials difficult. In addition, the absence of formal regulatory guidance on the design and conduct of antibiotic clinical trials in neonates and children has also hampered studies of clinical effectiveness. Our ability to compare therapies and treatment strategies would be improved by international collaboration to agree case definitions and outcome measures, similar to the collaboration that was effective in developing harmonized guidance for paediatric prescribing of antiretroviral drugs.^35^

### Dosing bands

The different age and weight dosing bands in leading global guidelines have been influenced by both practical considerations and historical usage. Although the most accurate method is to select the dose by weighing the child, this is not always possible. Simulations of orally administered amoxicillin in children show that the use of weight bands, rather than the exact weight, can often result in the administration of drug doses outside the therapeutic range.^14^ Perhaps the simplest method of dose selection is to use age as a proxy for weight, though in practice this is often the least accurate method. Nevertheless, it can be useful when the child’s recent weight is unknown, especially for drugs with a high therapeutic index (i.e. relatively safe drugs). Age bands need to be defined in a way that considers: periods of rapid weight change (e.g. during the first 6 months of life and around 8 to 9 years of age); the normal distribution of weight around the 50th percentile for specific ages; and local weight-for-age norms.^14^ Although there are theoretical concerns that underdosing can increase the risk of treatment failure (the therapeutic level may not be reached) or promote resistance selection, these risks can be proved only in large clinical trials. On the other hand, children who receive too high a dose may experience adverse events and discontinue treatment. Antibiotic-associated diarrhoea is the most common side-effect of penicillin in children, with oral penicillin, the rate is increased in younger children and there appears to be an association with higher doses.^36^ However, it remains unclear whether the child’s age or the drug’s formulation or dose is the main driver of severe diarrhoea.^36^

### Gaps in guidance

We identified several important omissions in global guidelines. First, we were unable to identify nationally endorsed, comprehensive treatment and dosing guidelines from Brazil, China, the Russian Federation or South Africa, although some individual institutions had their own local guidance. A unified approach within and, where appropriate, between countries would increase treatment standardization and make it easier to integrate the evidence. It is likely that many other countries, particularly low- and middle-income countries, also lack country-specific guidance and may have adopted recommendations developed elsewhere. In fact, published evidence often focuses on high-income settings, with little representation of low- or middle-income countries.^4^ However, guidelines developed in one country may not be appropriate for countries where the epidemiology and burden of infectious diseases is different. Second, further research is needed into clinical syndromes in which the antibiotic dose must be altered to take account of varying levels of drug resistance. Finally, it should be recognized that European, United States’ and WHO guidelines were all developed at a similar time and were all based on the same rapidly evolving evidence.

## Conclusions

The wide variation in paediatric antibiotic dosing recommendations we found between leading formularies could be rectified by: (i) carrying out a systematic review of the pharmacokinetic and clinical evidence underpinning current global guidance on the most commonly used antibiotics in children; (ii) producing an evidence-based, transparent, consensus, guidance document on the optimal dosing of Access antibiotics based on current knowledge; and (iii) identifying antibiotics that should be a priority for future research because more evidence is needed to optimize paediatric dosing, the nature of the evidence needed should be clear. Interested global parties could be brought together under the auspices of WHO’s antibiotic working group on the Model List of Essential Medicines for Children to develop an evidence-based consensus and identify research priorities.^3^
